# A systematic review and meta-analysis of community and primary-care-based hepatitis C testing and treatment services that employ direct acting antiviral drug treatments

**DOI:** 10.1186/s12913-019-4635-7

**Published:** 2019-10-28

**Authors:** Andrew Radley, Emma Robinson, Esther J. Aspinall, Kathryn Angus, Lex Tan, John F. Dillon

**Affiliations:** 1 NHS Tayside, Directorate of Public Health, Kings Cross Hospital, Clepington Road, Dundee, DD3 8EA UK; 2 University of Dundee, Division of Cardiovascular Medicines and Diabetes Ninewells Hospital and Medical School, Dundee, DD1 9SY UK; 3 School of Health and Life Sciences, Glasgow Caledonian University Glasgow and Health Protection Scotland, NHS National Services, Scotland, UK; 4 Institute for Social Marketing, Faculty of Health Sciences and Sport University of Stirling, Stirling, FK9 4LA UK

**Keywords:** Hepatitis C, Systematic review, Direct acting antiviral drugs, Primary care

## Abstract

**Background:**

Direct Acting Antiviral (DAAs) drugs have a much lower burden of treatment and monitoring requirements than regimens containing interferon and ribavirin, and a much higher efficacy in treating hepatitis C (HCV). These characteristics mean that initiating treatment and obtaining a virological cure (Sustained Viral response, SVR) on completion of treatment, in non-specialist environments should be feasible. We investigated the English-language literature evaluating community and primary care-based pathways using DAAs to treat HCV infection.

**Methods:**

Databases (Cinahl; Embase; Medline; PsycINFO; PubMed) were searched for studies of treatment with DAAs in non-specialist settings to achieve SVR. Relevant studies were identified including those containing a comparison between a community and specialist services where available. A narrative synthesis and linked meta-analysis were performed on suitable studies with a strength of evidence assessment (GRADE).

**Results:**

Seventeen studies fulfilled the inclusion criteria: five from Australia; two from Canada; two from UK and eight from USA. Seven studies demonstrated use of DAAs in primary care environments; four studies evaluated integrated systems linking specialists with primary care providers; three studies evaluated services in locations providing care to people who inject drugs; two studies evaluated delivery in pharmacies; and one evaluated delivery through telemedicine. Sixteen studies recorded treatment uptake. Patient numbers varied from around 60 participants with pathway studies to several thousand in two large database studies. Most studies recruited less than 500 patients. Five studies reported reduced SVR rates from an intention-to-treat analysis perspective because of loss to follow-up before the final confirmatory SVR test. GRADE assessments were made for uptake of HCV treatment (medium); completion of HCV treatment (low) and achievement of SVR at 12 weeks (medium).

**Conclusion:**

Services sited in community settings are feasible and can deliver increased uptake of treatment. Such clinics are able to demonstrate similar SVR rates to published studies and real-world clinics in secondary care. Stronger study designs are needed to confirm the precision of effect size seen in current studies. Prospero: CRD42017069873.

## Background

Of the 71 million persons infected with HCV, 5.6 million (8%) currently inject drugs [1, 2]. The World Health Organization (WHO) has defined global targets for HCV diagnosis and treatment, which represents a major step towards the aim of global elimination by 2030 [3].

However, rates of uptake of HCV testing, linkage to care and treatment remain low across many countries [4]. Barriers to accessing funded Direct Acting Antiviral (DAA) drug treatment may be due to provider concerns regarding co-morbidities, adherence, and side effects management [5]. Social factors affecting treatment access have been categorised as social stigma, housing, criminalisation, health care providers’ attitudes and stigmatising practices, and gender [6]. Individuals may prioritise other needs and may be wary of the consequences of a diagnosis on their circumstances; health systems may present complex and rigid arrangements that must be navigated in order to access care [7]. The stigma associated with both injecting drug use and HCV infection is pervasive [8]. The concept of the care cascade has focussed attention on the performance of different pathways and the attrition of patients accessing testing, diagnosis, treatment and care [9].

It is common in many developed and developing countries, for specialist clinicians to provide HCV treatment, often from hospital outpatient facilities [10]. Recently, prescribing of DAAs has become common practice in many countries [10]. Treatment of HCV with these medicines is simple and well-tolerated [11]. The safety profile and high efficacy of DAAs means that HCV treatment can be delivered by a range of non-specialist clinicians including nurses, pharmacists and general practitioners, therefore providing enhanced access to virological cure (SVR) [12]. The ease of transferring care to community and primary care environments is assisted by the use of treatment regimens that do not contain ribavirin or interferon [13]. Progress with implementing treatment pathways provided by non-specialists in community and primary care environments has been identified as one of the key steps in the elimination of HCV [14]. The World Health Organization’s Guidelines for the care and treatment of persons diagnosed with chronic hepatitis C virus infection promote simplified service delivery models: integration with other services; decentralised services supported by task-sharing; and community engagement, with the intention of reducing stigma and increase uptake of treatment [14].

This review was undertaken to identify rates of treatment uptake, treatment completion and achievement of sustained viral response for adults infected with hepatitis C using DAA-only treatment regimens in community and primary care-based care pathways, evaluated by studies using observational and experimental study designs. Studies that compared community-based treatment care pathways with specialist care were actively sought.

## Methods

This systematic review was undertaken and reported according to the Preferred Reporting Items for Systematic Reviews and Meta-Analyses (PRISMA) statement [15]. The methods of analysis and defined inclusion criteria were specified in advance and documented in a study protocol. The study was registered in PROSPERO (CRD42017069873). The PICOS elements defined for this review are set out in Table 1.

**Table 1:** Elements of the PICOS question defined for this review

	Inclusion	Exclusion
Population	Age 18 years and over Infected with hepatitis C	Age less than 18 years Co-infection with Hepatitis B virus Co-infection with HIV
Intervention	Provision of hepatitis C treatment in any primary care and community environments Treatment using any direct acting antiviral therapy Care provider could be any health care provider	Hepatitis C treatment in prison populations Treatment with ribavirin / interferon regimes as the primary intervention
Comparison	Care in any hospital or secondary care environment or no comparison group	
Outcome	Treatment uptake, treatment completion and SVR outcomes	
Study design	Observational studies, retrospective or prospective cohort studies, randomised trials; conference abstracts; qualitative and mixed methods studies	Case studies; systematic reviews

The rationale adopted in the design of the PICOS elements was intended to provide some answers to the questions raised by the WHO Guidance and its recommendations for simplified and decentralised treatment delivery models, integrated with other services in community and primary-care environments [14]. Therefore a population over 18 years old was selected, as being less likely to have gained their infection through vertical transmission. Co-infected individuals with other blood borne virus infections were also excluded as their care was likely to be more complex, requiring specialist rather than simplified care. Studies from prison populations were excluded since these individuals lived in contained communities. Studies that utilised interferon and ribavirin-based treatment regimes as the primary intervention were also excluded, since monitoring and patient management requirements, made simplified and decentralised care less likely. Sustained viral response at 12 weeks (SVR12) was taken as a marker for virological cure; failure to achieve SVR may be attributed to both treatment failure and loss to follow-up [16]. Studies were restricted to the English language since study resources precluded any translation activities. Published studies were utilised including conference abstracts, in order to capture results from early studies when the first DAAs were introduced into practice.

### Search strategy

Published research was identified by formal searches of five electronic databases (Cinahl, Embase, Medline, PsycINFO, PubMed) from January 2013 to December 2017, as well as Google Scholar. The last search was run on 11 December 2017. Search topics included “hepatitis C”, “treatment” and “setting”. A comprehensive list of search terms related to each of the search topics was used to develop a search strategy for each electronic database. Search strings were formulated by using a combination of keywords and indexed subject headings (MeSH and EMTREE terms). Primary care was defined using the WHO accepted terminology that promotes Primary Care as a key process in the health system: “it is first-contact, accessible, continued, comprehensive and coordinated care” [17] and community environments being the geographical locations where groups of people live.

The full search strategy is set out in Additional file 1. Reference lists of selected articles, citing articles and relevant review articles retrieved during the initial search were hand-searched and forward citation checks were undertaken to identify any additional studies. Abstracts from the selected scientific conferences were screened for review eligibility.

### Study selection

Data retrieved through the study search strategy were imported into EndNote X8 (Thomson Reuters, New York, NY, USA) and any duplicates removed. Titles obtained from the initial search strategy were screened and irrelevant citations were removed. Abstracts were then assessed using the inclusion and exclusion criteria by two reviewers independently (AR and LT) to establish a relevant pool of evidence for further evaluation. Full-texts from all abstracts identified for further evaluation and were double-screened independently by the two reviewers to assess whether they met the defined inclusion and exclusion criteria. In the event of a disagreement, the senior investigator (JFD) determined final inclusion. The lead author contacted conference abstract authors to attempt to obtain further study results if available. Studies published from identified conference abstracts were screened for review.

### Data collection process and data items collected

Data from studies included for analysis were extracted by the lead author (AR) using a standardised data extraction form (Microsoft Excel 2010 Redmond, WA, USA). A second reviewer (ER) also independently assessed the extracted data, and disagreements were resolved by discussion until consensus was reached. The following variables were documented: first author, title, publication year, study design, study location, setting, intervention description, comparator description, sample size outcome description and number of participants achieving SVR12 (and percentage if applicable).

### Risk of bias assessment in individual studies

The risk of bias in individual studies was assessed by two reviewers (AR and ER) using the Cochrane Collaboration’s risk of bias tool for randomised studies [18] and the “Newcastle-Ottawa Scale (NOS) for assessing the quality of nonrandomised studies in meta-analyses” [19]. For randomised studies, these outcomes were evaluated along the six domains: selection bias, performance bias, detection bias, attrition bias, reporting bias, and other bias. The domains deemed as ‘high risk’ of bias for each study per outcome were determined. Outcomes for the non-randomised studies were evaluated along seven domains: bias due to confounding; bias in selection of participants into study; bias in classification of interventions; bias due to deviations from intended interventions; bias due to missing data; bias in measurement of outcomes; and bias in selection of the reported result. The overall risk of bias for these studies was classified into five categories: low risk of bias; moderate risk of bias; serious risk of bias; critical risk of bias or no information.

The NOS scale measures three items: selection of cases and controls including their definition and representativeness; comparability of cases and controls in design and analysis; and exposure ascertainment. The scale has a minimum score of 0 and a maximum score of 9. Risk of bias was rated as high, medium or low according to the scores obtained by reviewing the selection, comparator and exposure categories. Risk of bias was rated low if studies scored 8 or 9; medium risk if studies were scored as 6 or 7. Studies were rated as having a high risk of bias if they were scored as having 5 or less or scored zero for the comparator category [20].

We assessed the strength of evidence using GRADE [21]. The scheme evaluates a required group of domains (study limitations, directness, consistency, precision and reporting bias) and enables grading of the strength of evidence as High; Moderate; Low or Insufficient. Use of this approach enabled us to summarise the outcomes and findings and make clear judgements about the effects of the interventions.

### Data analysis

The characteristics and findings of the studies included were summarised and structured using tables. Studies evaluating similar service environments in community and primary care-settings were grouped together to facilitate comparison.

Study designs, participants, interventions and reported outcomes varied significantly, and a meta-analysis was unable to be performed on all included studies. Studies were excluded from the meta-analysis if the reviewers considered them to be sufficiently flawed so as not to contribute meaningfully to the body of evidence [21].

The characteristics and findings of included studies amenable to meta-analysis were summarised using tables and forest plots. Risk ratio (RR) and corresponding 95% confidence interval (95% CI) was calculated for each study outcome, using the initial number of eligible participants included and the number achieving the outcome of interest in each arm. Analyses were conducted using statistical package Stata v14.0 (College Station, TX, USA).

### Data synthesis

#### Deriving pooled estimates of treatment uptake, treatment completion and SVR

Treatment uptake, treatment completion and SVR and their exact 95% confidence intervals (CIs) were calculated assuming a binomial distribution. Pooled estimates were derived using random- or fixed-effects methods, according to whether significant heterogeneity (defined as I^2^ > 30%) was or was not present, respectively. Sensitivity analysis was used to assess the impact of study quality (restricting to studies with an NOS score ≥ 6) on the pooled estimate of SVR.

Further sensitivity analysis was used to assess the impact of conference abstracts on the pooled estimate of SVR. We identified studies using similar environments from which to deliver care and grouped them into categories. Factors identified as linking studies within categories were examined as well as factors that differentiated studies from each other.

## Results

### Study selection

**Fig. 1:**
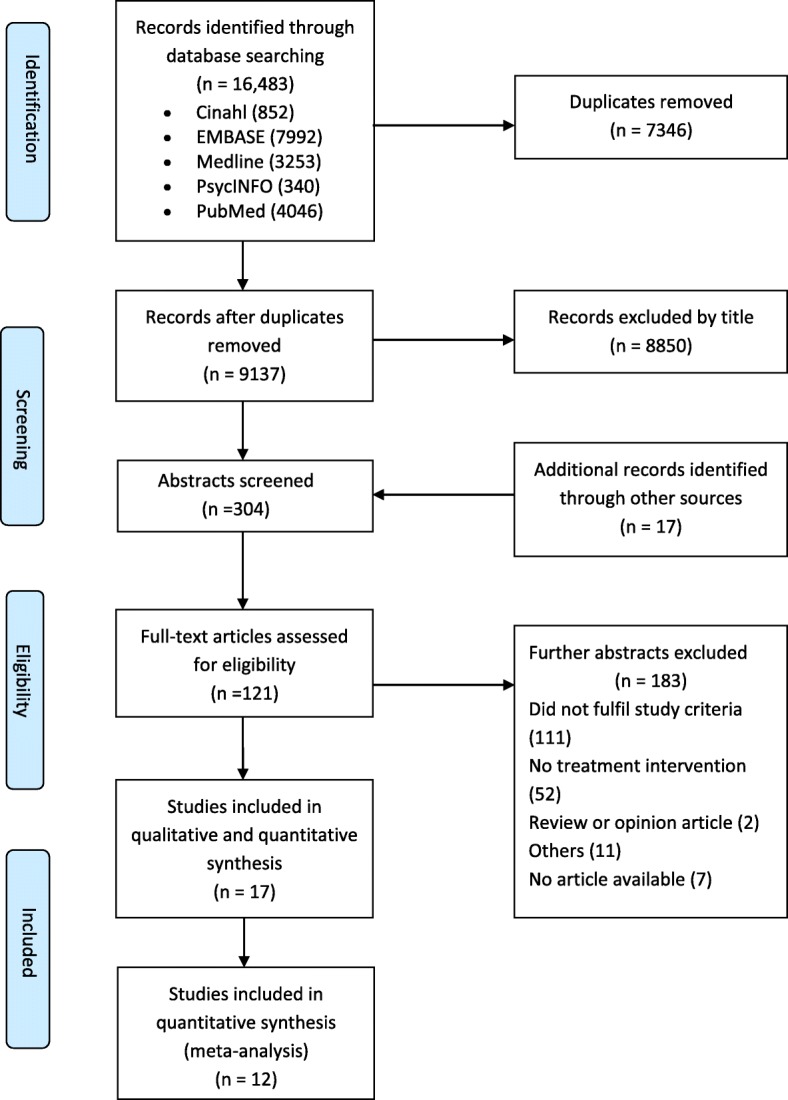
Flow diagram of search results

The searches yielded 9137 publications after removal of duplicates (Fig. 1). This resulted in 121 articles retrieved for full text inspection and 17 included for analysis. Explanations for exclusion of studies at the full text stage are provided in Fig. 1. These included: did not fulfil inclusion criteria; no treatment intervention; review or opinion article; other (e.g. insufficient detail reported in conference abstract).

### Study characteristics

Studies evaluated care pathways in primary care [22–28]; in integrated health systems (Extension for Community Healthcare Outcomes, ECHO) [29–32]; in opioid treatment centres [33–35]; in pharmacies/pharmacist clinics [36, 37] and by telemedicine [38]. Characteristics and findings of included studies are set out in Table 2. These studies originated from United States of America (8); Australia (5); United Kingdom (2); and Canada (2). The number of identified studies published as conference abstracts reflected the length of time that DAAs have been widely available outside specialist environments. Six from seventeen studies were only available as conference abstracts. There were two randomised controlled trials, four cohort studies, nine retrospective data analyses and two prospective non-experimental designs. All were conducted on populations at high risk of HCV infection, such as people who inject drugs and people on Opioid Substitution Therapy (OST) programmes. Table 3 describes the outcomes from the meta-analysis of selected studies and Table 4 defines the Strength of Evidence Assessment for identified studies answering the PRISMA objective. Details of assessment of bias and design for studies are located in Additional file 2 (non-randomised) and Additional file 3 (randomised)).

**Table 2:** Characteristics and findings of included studies

Care Location	Year	Country	Design	Intervention	Comparator	Number of participants	Uptake (%)	SVR (%)
Primary Care								
Bloom	2017	Australia	Prospective cohort study of treatment uptake and SVR	Adherence to DAA treatment protocols	Treatment by tertiary care provider	1044	503 (40.6)	253 (50.2)
Francheville	2017	Canada	Prospective observational study design	Specialist nurse-led care	No comparator group	242	93 (38.4)	82 (88.2)
Kattakuzhy	2017	USA	Non-randomised open label study	Treatment by primary care providers (PCP) and nurse practitioners (NP)	Standard care - Treatment by secondary care clinic	NP 150 PCP 160		NP 134 (89.3) PCP139(86.9)
McCLure	2017	Australia	Retrospective data analysis of SVR12	Nurse-led care and GP remote consultation	Specialist care in Tertiary centre	Nurse-led 70	50 (74.3)	46 (65.7)
Miller	2016	USA	Retrospective observational study	Treatment by primary care providers	No comparator group	95		79 (83)
Norton	2017	USA	Retrospective cohort study of SVR	Treatment in urban primary care centre	SVR 12 in PWIDs and non_PWIDs	89		85(95.5)
Wade	2018	Australia	Randomised controlled trial	Testing, assessment and treatment in primary care	Testing, assessment and treatment in tertiary care	59	31 (52.5)	14 (23.7)
Integrated Health Systems (ECHO)								
Abdulameer	2016	USA	Retrospective data analysis of SVR 12	VA-Echo model supporting primary care providers	No comparator group	588		318 (54)
Beste	2017	USA	Retrospective cohort study of treatment uptake and SVR	VA-Echo model supporting primary care providers	Standard care - Treatment by unexposed primary care providers	6431	1303 (21.4)	(58.2)
Buchanan	2015	United Kingdom	Retrospective data analysis	Community-based outreach clinic	Standard care - Treatment by secondary care clinic	77	24 (31.2)	
Georgie	2016	USA	Retrospective data analysis of SVR12	VA-Echo model supporting primary care providers	Treatment by sub-specialist providers	623		Genotype 1 (GT1) (99) GT2 (98) GT3 (79)
Opioid Treatment Centres								
Butner	2017	USA	Retrospective data analysis	Opioid treatment programme	No comparator group	75	75.0	64 (85.0)
Morris	2017	Australia	Retrospective data analysis of treatment uptake and SVR	Treatment in a community-based harm reduction and treatment facility	No comparator group	127	122 (96)	102 (80.3)
Read	2017	Australia	Retrospective data analysis of SVR12	Treatment of PWIDs in primary care setting	No comparator group	72		59 (81.9)
Pharmacies / Pharmacist Clinics								
David	2017	USA	Retrospective data analysis of SVR12	Pharmacy-managed clinics	Treatment by non-pharmacist providers	204		(83.6)
Radley	2017	United Kingdom	Pilot cluster RCT of treatment uptake and SVR	Treatment in community Pharmacy	Treatment by secondary care clinic	26	3 (11.5)	3 (11.5)
Telemedicine								
Cooper	2017	Canada	Retrospective cohort study of treatment uptake and SVR	Use of telemedicine	Treatment by secondary care clinic	157	35.0	18 (11.5)

**Table 3:** Meta-analysis of studies examining treatment uptake, treatment completion and SVR among people with Hepatitis C treated in a variety of community settings or specialist hospital care

Inclusion Criteria	Treatment Uptake			Treatment Completion			SVR		
	No. Of studies	Heterogeneity (I^2^)	Pooled estimate (95% CI)	No. Of studies	Heterogeneity (I^2^)	Pooled estimate (95% CI)	No. Of studies	Heterogeneity (I^2^)	Pooled estimate (95% CI)
Opioid Treatment Centres				2	77.7%	91.9 (82.2–100)	3	0.0%	82.3 (77.8–86.8)
Integrated Health System (ECHO)	1	Not applicable	75.6 (68.0–83.2)	1	Not applicable	96.8 (93.2–100)	2	84.6%	81.3 (66.9–95.5)
Telemedicine	1	Not applicable	22.3 (15.8–28.8)				1	Not applicable	51.4 (34.8–68.0)
Primary Care	1	Not applicable	67.4 (53.9–80.9)	1	Not applicable	100 (97.95–100)	5	94.9%	74.4 (60.3–88.5)
Pharmacies / Pharmacist Clinics	1	Not applicable	66.67 (58.3–75.1)				2	89.0%	79.0 (79.2–98.9)
Specialist Care	2	0.0%	34.5 (31.79–37.29)				5	96.8%	73.46 (60.9–85.9)

Abbreviations: *CI*, Confidence interval; *SVR*, Sustained virologic response

a. Random-effects method used if I^2^ ≥ 30%

**Table 4:** Summary of key findings, outcomes and strength of evidence

Outcome	Study designs/ No. Studies	Findings and Direction of Effect	GRAD E[21]
1. Uptake of HCV treatment	RCT – 2 Cohort – 3 Observational – 5	Two RCTs assessed as having low risk of bias reported a positive effect on uptake with precision and a consistent positive direction of effect. One cohort study assessed as having medium-grade study limitations also reported a positive effect on uptake.	Medium
2. Completion of Treatment	Cohort - 1 Observational - 2	One cohort study with medium study limitations reported a positive direction of effect on uptake.	Low
3. Sustained Viral Response at 12 weeks (%)(SVR12)	RCT −2 Cohort - 4 Observational - 11	Two RCTs assessed as having low risk of bias reported a positive effect on SVR but were imprecise in the estimate of effect size. Four cohort studies and 11 observational studies with over 10,000 participants all reported a consistent positive direction of effect, but with significant study limitations.	Medium

### Primary care

Seven studies evaluated interventions to enhance treatment uptake and achievement of SVR in primary care environments [22–28]. One study was a randomised controlled trial (RCT), two were cohort studies and four were non-randomised studies. Four studies utilised nurses in delivery of the care pathway. Three studies included uptake of testing and assessment in their description of care and all the studies discussed uptake of treatment and ascertainment of SVR. The RCT reported a significant difference between those commencing treatment in primary care arm than in the Standard of Care arm (SOC) (75% Vs 34%, *p* < 0.001) and proportion gaining an SVR12 was significantly higher in the primary care arm than in the SOC arm (49% vs 34%, *p* = 0.043).

Two studies reported a reduction in potential SVR rates because of failure of participants to complete the confirmatory blood test at 12 weeks after completion of DAA treatment. All studies reported increased access to treatment in primary care environments and high rates of SVR attainment.

### Integrated health systems (ECHO)

Four studies provided evaluations of care through integration of specialist centres with primary care delivery [29–32]. One study was a retrospective cohort study and three were non-randomised studies. Three of the four studies utilised the “ECHO” care pathway in which hepatitis specialists support primary care providers through video-conferencing and collaboration on specific cases, with a defined curriculum and active mentorship [39]. None of the studies discussed uptake of testing amongst their treated cohorts. All studies increased access to treatment and high rates of attainment of SVR.

### Opioid treatment Centres

Three studies evaluated care provision in dedicated setting where people with opioid addiction received harm reduction and treatment services [33–35]. All three studies were non-randomised analyses of treatment data and assessed the uptake and completion of treatment by participants using these services. No assessment of the extent of testing of these populations was discussed. All studies reported high rates of treatment uptake and treatment completion in diagnosed individuals. These studies all described problems with retention of participants in the service post-treatment with consequent reductions in uptake of confirmatory SVR testing.

### Pharmacies / pharmacist clinics

Two studies evaluated hepatitis C care provision by pharmacists in community and primary care settings [36, 37]. One study was a feasibility RCT that compared the delivery of a community pharmacy test and treatment pathway with standard hospital-based care. One study was a non-randomised data analysis. The RCT demonstrated an increase in testing uptake, when the participant received all care in a pharmacy environment and showed increased retention in care. Data from this study also demonstrates a marked loss of patients from the care pathway when they were asked to attend the local hospital. The non-randomised study concluded that patients treated in pharmacist clinics achieve high rates of SVR similar to non-pharmacist clinics.

### Telemedicine

A single cohort database study [38] compared treatment uptake and SVR rates in participants cared for through a telemedicine pathway (*n* = 157) with participants cared for through a standard care pathway (*n* = 1130). The study demonstrated increased access to care form under-served and remote areas and concluded that the telemedicine intervention achieved high rates of treatment initiation and SVR.

### Data synthesis

**Fig. 2:**
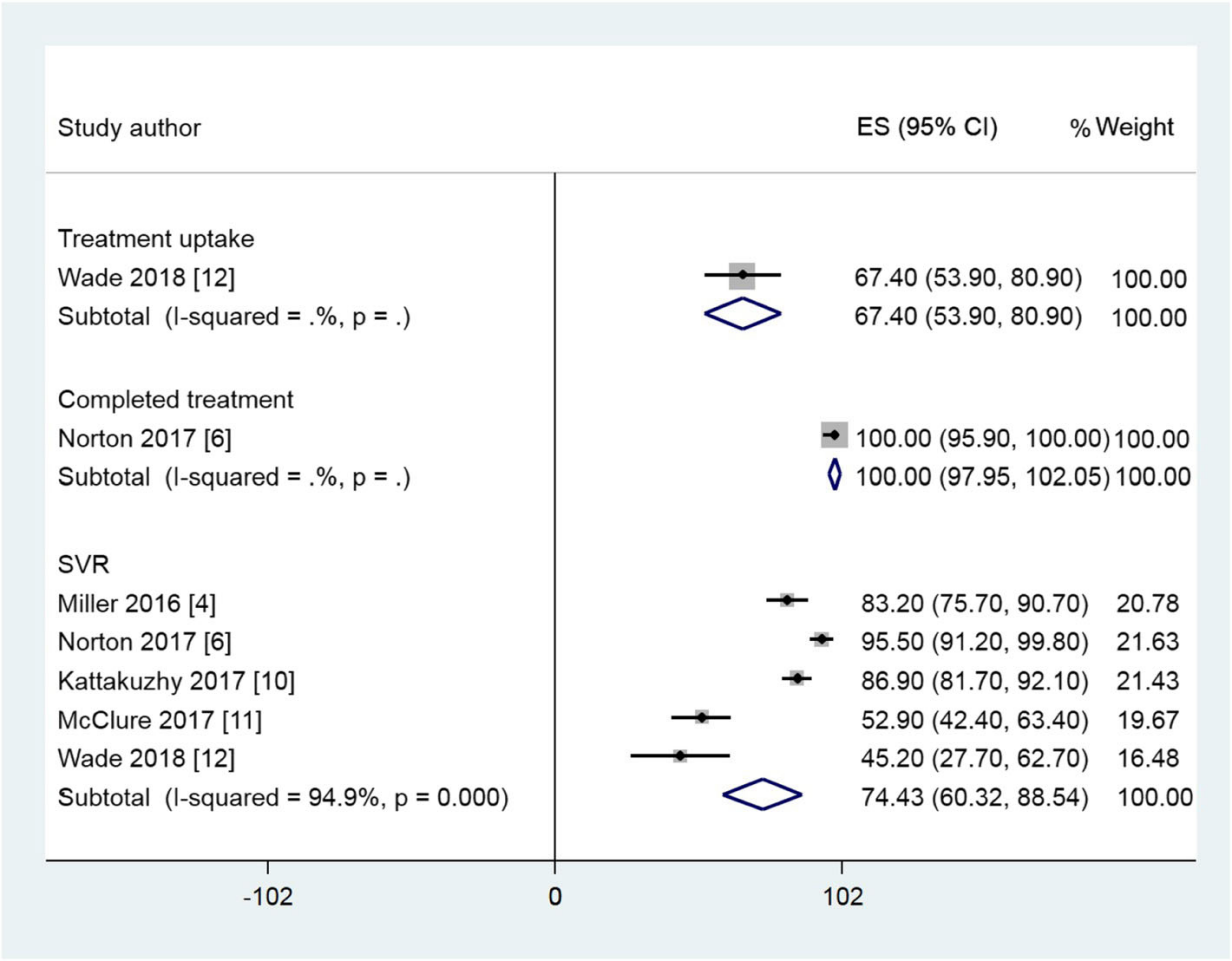
Forest plots of treatment uptake, completed treatment and SVR rates for selected studies in the Primary Care Location

**Fig. 3:**
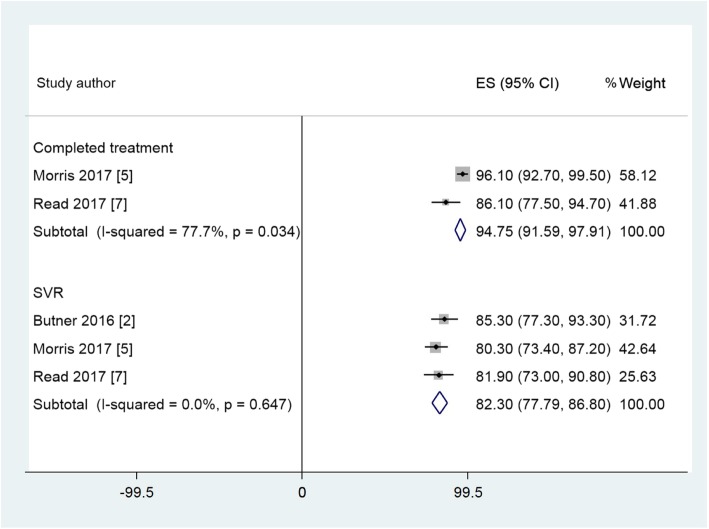
Forest plots of completed treatment and SVR rates for selected studies in Opioid Treatment Centres Location

**Fig. 4:**
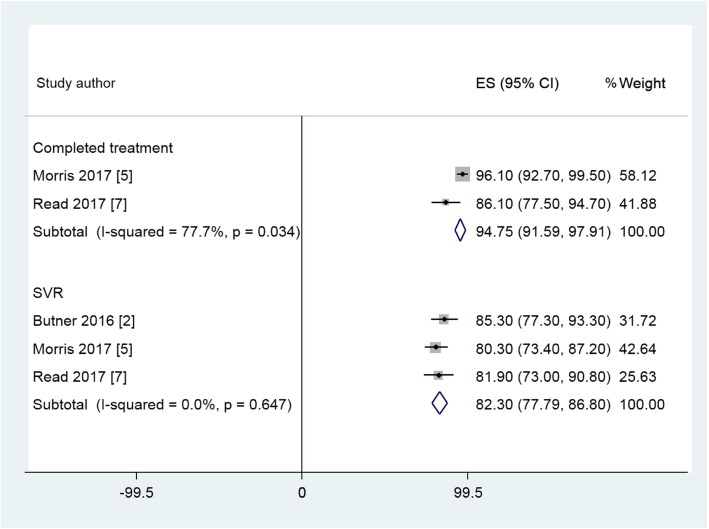
Forest plots of treatment uptake and SVR rates for studies in the Pharmacy / Pharmacist Clinic Location

The 12 studies eligible for meta-analysis examined treatment uptake, completion and SVR in a variety of primary care environments; integrated systems (ECHO) that linked specialists with primary care providers; opioid treatment centres; pharmacies / pharmacist clinics; telemedicine and specialist hospital care. The remaining five studies were unsuitable for meta-analysis due to non-reporting of the required outcomes, use of Pegylated interferon or insufficient time to achieve SVR. Across the 12 studies, the pooled estimate is shown in Additional file 4 Table S3. Forest plots for suitable studies are set out in Figs. 2, Fig. 3 and Fig. 4. These plots demonstrate that across the variety of community and primary care environments, a consistent direction of effect to improve treatment uptake, treatment completion and achievement of SVR is seen. Greater uptake was seen for the Primary Care and Pharmacy Locations, compared to the Specialist Care Location and comparable SVR rates were demonstrated (Table 2).

In this analysis, heterogeneity was noted to be high so a sensitivity analysis restricting to higher-quality studies (NOS score ≥ 6) was performed. Despite this the heterogeneity remained high. A further sensitivity analysis was performed restricting the meta-analysis to published studies only. See Additional file 3 in the appendix. This had no impact on heterogeneity.

## Discussion

This paper reviews evaluations of care pathways that utilise DAAs in a range of community and primary care settings. The WHO Guidelines on care and treatment of persons diagnosed with chronic HCV infection promote simplified service delivery models; integration with other services; decentralised services supported by task-sharing; and community engagement to address stigma and increase reach [14]. The studies considered in this systematic review and meta-analysis therefore provide some evidence for the extent of implementation of these guidelines.

The studies identified that met our inclusion criteria were grouped according to location: primary care; integrated health care systems (ECHO); opioid treatment centres; in pharmacies / pharmacist clinics; and through telemedicine. These care pathways acknowledged the need to provide local services with reach into the communities where people with hepatitis C live their lives.

In all three areas assessed in our study: uptake of treatment; completion of treatment; and attainment of SVR, a positive outcome was reported by all identified studies. This was seen across each of the distinct environments from which the care was provided. Since the positive outcomes were drawn from distinctly different pathways of care, further confidence might be inferred from this consistency of direction of effect. However, amongst the studies that met our inclusion criteria, there was a lack of studies using comparators from specialist centres. Data contained in these studies nevertheless demonstrated high uptake of treatment and high rates of attainment of SVR: among populations of vulnerable people who normally struggle to access care. Studies that did include comparators showed no significant differences in uptake or SVR. Several of the studies reported an increased uptake of treatment, but most reported equivalence. Some studies reported lower rates of attainment of SVR, because of study participants failing to undergo a confirmatory blood test post-treatment, within the study timelines. With DAAs SVR rates of greater than 97% are delivered if patients adhere to treatment, therefore completion of therapy can be a surrogate for SVR [16].

Previous systematic reviews have considered barriers and facilitators to care, as well as the views and experiences of people who inject drugs [7, 40]. These studies concluded that the target groups for HCV often had poor levels of knowledge about the infection and of the processes involved with testing and treatment. A fear of stigma and discrimination and a reticence to discuss risk behaviours tended to prevent engagement. These barriers could be addressed through educating participants, increasing awareness and redress of institutionalised stigma and integrating HCV treatment pathways into other services where the target group were likely to go.

Increased uptake of testing has been observed when testing is offered at the same time as other routine care [4]; with integrated services for both opioid users and with mental health services. There are advantages to targeting services at populations with predicted high prevalence of HCV [41]. Provision of HCV treatment as part of a directly observed treatment arrangement, increased attainment of SVR [42]. Achievement of these factors within local health systems needs to be commonplace if the WHO target for elimination is to be met [43]. There is some evidence that this is now happening [44].

The results from this systematic review highlight the lack of well-controlled randomised controlled trials and comparative studies, with just two randomised controlled trials identified and four cohort studies. While the publication of such studies is an important step in building confidence that decentralisation of hepatitis C treatment can be accomplished, the paucity of evidence reflects the difficulty in funding pathways to care studies and the relatively recent removal of the restrictions on the use of DAAs. Two further studies have been commenced identify that further evaluations of interferon-free treatments in primary care environments are underway [45, 46].

As with most systematic reviews, the quality of the studies and the heterogeneity of the study populations included in the analysis present a limitation of this study. The sensitivity analyses performed for our analysis did not have an impact on heterogeneity, meaning that an unexplained source of heterogeneity may be present. These difficulties may reflect the variety of ways in which patients can access HCV treatment. This may be positive and may be explained by the development of more patient centred pathways. These factors prevented a meta-analysis being achieved for many of the studies identified as eligible through the PICOS question defined for this review. Many of the studies that met the inclusion criteria were only available as conference abstracts at the time of review, including one of the randomised controlled trials. Nevertheless, over 10,000 participants were included in the identified studies. All studies had a consistent direction of effect, providing optimism that future evaluations will confirm with precision the effect size that should be delivered by simplifying treatment pathways and decentralising them to primary care. In terms of further limitations, we acknowledge limitations in the chosen methods for the systematic review, including potential publication bias to the findings by excluding non-English language studies; or any other biases introduced by our chosen inclusion and exclusion criteria.

## Conclusion

This systematic review and meta-analysis identified studies which demonstrate the feasibility of decentralising care and providing local services with reach into communities of people infected with HCV. Such pathways may increase uptake of treatment and can provide sustained viral responses equivalent to those attained in specialist centres. Further studies are needed to confirm the promising start to the implementation of interferon-free treatment regimens. The successful implementation of such pathways to deliver successful patient outcomes is a key requirement for a “treatment as prevention” strategy as a pathway to elimination of HCV [47].

## Supplementary information

**Additional file 1.** Sample search strategy for MEDLINE(R) Epub Ahead of Print, In-Process & Other Non-Indexed Citations, Ovid MEDLINE(R) Daily and Ovid MEDLINE(R) 1946 to Present

**Additional file 2.** Table S1 Assessment of risk of bias for included studies – Newcastle/Ottawa Assessment non-randomised studies.

**Additional file 3.** Table S2 Cochrane Assessment of Randomised Studies.

**Additional file 4.** Table S3Meta-analysis of published studies examining sustained virologic response among people with Hepatitis C treated in a variety of community settings or specialist hospital care.

## Abbreviations

DAA: Direct Acting Antiviral
GRADE: Grading of Recommendations Assessment, Development and Evaluation
HCV: Hepatitis C
HIV: Human Immunodeficiency Virus
NOS: Newcastle Ottawa Scale
OST: Opioid Substitution Therapy
PICOS: Population; Intervention; Comparison; Outcome; Study Design
PRISMA: Preferred Reporting Items for Systematic Reviews and Mata-Analysis
PWID: People Who Inject Drugs
RCT: Randomised Controlled Trial
RR: Risk Ratio
SOC: Standard of Care
SVR: Sustained Viral Response
SVR12: Sustained Viral response at 12 weeks
WHO: World Health Organization
