# Supplementary material for: A systematic review and meta-analysis of community and primary-care-based hepatitis C testing and treatment services that employ direct acting antiviral drug treatments
Source: BMC Health Serv Res. 2019 Oct 28;19:765. doi: 10.1186/s12913-019-4635-7 (PMC6819346; doi:10.1186/s12913-019-4635-7)
Supplement: Supplementary file 1 — Additional file 1. Sample search strategy for MEDLINE(R) Epub Ahead of Print, In-Process & Other Non-Indexed Citations, Ovid MEDLINE(R) Daily and Ovid MEDLINE(R) 1946 to Present [file 12913_2019_4635_MOESM1_ESM.docx]

Supplementary file 1.

Sample search strategy for MEDLINE(R) Epub Ahead of Print, In-Process & Other Non-Indexed Citations, Ovid MEDLINE(R) Daily and Ovid MEDLINE(R) 1946 to Present

| \| # \| Searches \| \| --- \| --- \| \| 1 \| exp Hepatitis C/ \| \| 2 \| exp Hepatitis C, Chronic/ \| \| 3 \| exp Hepatitis C Antibodies/bl \| \| 4 \| Hepacivirus/ \| \| 5 \| Hepatitis C.mp. \| \| 6 \| hepatitic C.mp. \| \| 7 \| Direct Acting Antiviral.mp. \| \| 8 \| Direct-Acting Antiviral.mp. \| \| 9 \| Antiviral Agents/ \| \| 10 \| ("hepatitis C" or HCV).mp. \| \| 11 \| 9 and 10 \| \| 12 \| 1 or 2 or 3 or 4 or 5 or 6 or 7 or 8 or 11 \| \| 13 \| treatment*.mp. \| \| 14 \| family.mp. \| \| 15 \| general.mp. \| \| 16 \| local.mp. \| \| 17 \| regional.mp. \| \| 18 \| walk-in.mp. \| \| 19 \| communit*.mp. \| \| 20 \| primary.mp. \| \| 21 \| outreach.mp. \| \| 22 \| maternal.mp. \| \| 23 \| GP.mp. \| \| 24 \| GPs.mp. \| \| 25 \| dentist$1.mp. \| \| 26 \| healthcentre$1.mp. \| \| 27 \| health centre$1.mp. \| \| 28 \| healthcenter$1.mp. \| \| 29 \| health center$1.mp. \| \| 30 \| healthcare.mp. \| \| 31 \| health care.mp. \| \| 32 \| pharmacy.mp. \| \| 33 \| pharmacies.mp. \| \| 34 \| pharmacist$1.mp. \| \| 35 \| Opiate Substitution Treatment/ \| \| 36 \| methadone.mp. \| \| 37 \| buprenorphine.mp. \| \| 38 \| ((opioid or opiate) adj1 (substitution or replacement)).mp. \| \| 39 \| Remote Consultation/ \| \| 40 \| Telerehabilitation/ \| \| 41 \| telemedicine.mp. \| \| 42 \| telehealth.mp. \| \| 43 \| teleconsultation$1.mp. \| \| 44 \| accessibil*.mp. \| \| 45 \| marginal*.mp. \| \| 46 \| underserved.mp. \| \| 47 \| under-served.mp. \| \| 48 \| 14 or 15 or 16 or 17 or 18 or 19 or 20 or 21 or 22 or 23 or 24 or 25 or 26 or 27 or 28 or 29 or 30 or 31 or 32 or 33 or 34 or 35 or 36 or 37 or 38 or 39 or 40 or 41 or 42 or 43 or 44 or 45 or 46 or 47 \| \| 49 \| 12 and 13 and 48 \| \| 50 \| ..l/ 49 yr=2013-2018 \| |
| --- | --- | --- | --- | --- | --- | --- | --- | --- | --- | --- | --- | --- | --- | --- | --- | --- | --- | --- | --- | --- | --- | --- | --- | --- | --- | --- | --- | --- | --- | --- | --- | --- | --- | --- | --- | --- | --- | --- | --- | --- | --- | --- | --- | --- | --- | --- | --- | --- | --- | --- | --- | --- | --- | --- | --- | --- | --- | --- | --- | --- | --- | --- | --- | --- | --- | --- | --- | --- | --- | --- | --- | --- | --- | --- | --- | --- | --- | --- | --- | --- | --- | --- | --- | --- | --- | --- | --- | --- | --- | --- | --- | --- | --- | --- | --- | --- | --- | --- | --- | --- | --- | --- |
