# Supplementary material for: A systematic review and meta-analysis of community and primary-care-based hepatitis C testing and treatment services that employ direct acting antiviral drug treatments
Source: BMC Health Serv Res. 2019 Oct 28;19:765. doi: 10.1186/s12913-019-4635-7 (PMC6819346; doi:10.1186/s12913-019-4635-7)
Supplement: Supplementary file 2 — Additional file 2. Table S1 Assessment of risk of bias for included studies – Newcastle/Ottawa Assessment non-randomised studies. [file 12913_2019_4635_MOESM2_ESM.docx]

Supplementary Table 1: Assessment of risk of bias for included studies – Newcastle/Ottawa Assessment non-randomised studies

| **Study** | **Design** | **Assessment of Bias** | | | **Comments** |
| --- | --- | --- | --- | --- | --- |
|  |  | **Selection** | **Comparability** | **Outcome** |  |
| Abdulameer | Retrospective data analysis of SVR 12 | 4 | 0 | 1 | Conference abstract |
| Beste | Retrospective cohort study of treatment uptake and SVR12 | 4 | 2 | 3 |  |
| Bloom | Prospective cohort study of treatment uptake and SVR 12 | 4 | 2 | 3 | Conference abstract |
| Buchanan | Retrospective data analysis | 3 | 1 | 1 | Conference abstract |
| Butner | Retrospective data analysis | 4 | 2 | 3 |  |
| Cooper | Retrospective cohort study of treatment uptake and SVR | 4 | 2 | 3 |  |
| David | Retrospective data analysis of SVR12 | 4 | 1 | 2 | Conference Abstract |
| Francheville | Prospective observational study design | 2 | 0 | 1 |  |
| Georgie | Retrospective data analysis of SVR12 | 4 | 2 | 2 | Conference Abstract |
| Kattakuzhy | Non-randomised open label study | 3 | 0 | 3 |  |
| McCLure | Retrospective data analysis of SVR12 | 4 | 0 | 2 | Conference abstract |
| Miller | Retrospective observational study | 3 | 0 | 3 |  |
| Morris | Retrospective data analysis of treatment uptake and SVR 12 | 3 | 0 | 1 |  |
| Norton | Retrospective cohort study of SVR 12 | 3 | 1 | 3 |  |
| Read | Retrospective data analysis of SVR12 | 3 | 0 | 3 |  |
