# Supplementary material for: A systematic review and meta-analysis of community and primary-care-based hepatitis C testing and treatment services that employ direct acting antiviral drug treatments
Source: BMC Health Serv Res. 2019 Oct 28;19:765. doi: 10.1186/s12913-019-4635-7 (PMC6819346; doi:10.1186/s12913-019-4635-7)
Supplement: Supplementary file 3 — Additional file 3. Table S2 Cochrane Assessment of Randomised Studies. [file 12913_2019_4635_MOESM3_ESM.docx]

Supplementary Table 2: Cochrane Assessment of Randomised Studies

| Radley | Pilot cluster RCT of treatment uptake and SVR 12 | **R** | **A** | | | **S** | | **O** | | **B_p_** | | **B_o_** | | **I** |  |
| --- | --- | --- | --- | --- | --- | --- | --- | --- | --- | --- | --- | --- | --- | --- | --- |
|  |  | L | L | | | L | | L | | L | | L | | L |  |
| Wade | Randomised Controlled Trial of treatment uptake and SVR 12 | **R** | | **A** | **S** | | **O** | | **B_p_** | | **B_o_** | | **I** | | Conference abstract |
|  |  | L | | L | L | | L | | L | | L | | L | |  |
