# Supplementary material for: A systematic review and meta-analysis of community and primary-care-based hepatitis C testing and treatment services that employ direct acting antiviral drug treatments
Source: BMC Health Serv Res. 2019 Oct 28;19:765. doi: 10.1186/s12913-019-4635-7 (PMC6819346; doi:10.1186/s12913-019-4635-7)
Supplement: Supplementary file 4 — Additional file 4. Table S3Meta-analysis of published studies examining sustained virologic response among people with Hepatitis C treated in a variety of community settings or specialist hospital care. [file 12913_2019_4635_MOESM4_ESM.docx]

Supplementary Table 3: Meta-analysis of published studies examining sustained virologic response among people with Hepatitis C treated in a variety of community settings or specialist hospital care.

| Inclusion Criteria | SVR | | |
| --- | --- | --- | --- |
|  | No. Of studies | Heterogeneity^a^  (I^2^) | Pooled estimate (95% CI) |
| Places where PWIDs are Treated | 3 | 0.0% | 82.3 (77.8-86.8) |
| Community outreach | 1 | Not applicable | 88.2 (81.6 – 94.8) |
| Telemedicine | 1 | Not applicable | 51.4 (34.8-68.0) |
| Primary care | 3 | 81.3% | 88.9 (81.6 – 96.3) |
| Pharmacy | 1 | Not applicable | 93.8 (88.5 – 99.1) |
| Specialist care | 2 | 97.8% | 72.1 (49.9 – 94.2) |
